# Inflammatory Bowel Diseases in the Elderly: A Focus on Disease Characteristics and Biological Therapy Patterns

**DOI:** 10.3390/jcm13102767

**Published:** 2024-05-08

**Authors:** Renata Talar-Wojnarowska, Miłosz Caban, Marta Jastrzębska, Małgorzata Woźniak, Aleksandra Strigáč, Ewa Małecka-Wojciesko

**Affiliations:** 1 Department of Digestive Tract Diseases, Faculty of Medicine, Medical University of Lodz, 90-153 Lodz, Poland; renata.talar-wojnarowska@umed.lodz.pl (R.T.-W.); mmw89@op.pl (M.W.); aleksandra.strigac@gmail.com (A.S.); ewa.malecka-panas@umed.lodz.pl (E.M.-W.); 2 Department of Biochemistry, Faculty of Medicine, Medical University of Lodz, 92-215 Lodz, Poland; 3 Department of Gastroenterology, Health Care Center, 26-200 Konskie, Poland; marta.bar@interia.pl

**Keywords:** adverse effects, biologics, Crohn’s disease, inflammatory bowel diseases, safety, ulcerative colitis

## Abstract

**Background**: The incidence of inflammatory bowel diseases (IBDs) in elderly patients is constantly increasing. It results from the combination of an aging population with compounding prevalence of IBD, as well as the growing burden of elderly-onset IBD. The clinical characteristics of elderly patients differ from young subjects with IBD due to the multimorbidity or polypharmacy, affecting the choice of adequate therapeutic options. The aim of this study was to determine the clinical aspects and biological therapy safety in elderly Polish IBD patients. **Methods**: We conducted a retrospective study aimed at describing the demographic, clinical, and management characteristics of IBD patients treated with a biological therapy in two referral centers within the National Drug Program in Poland. **Results**: Out of the entire group of 366 studied patients, 51 (13.9%) were aged over 60—32 with ulcerative colitis (UC) and 19 with Crohn’s disease (CD). The disease location was predominantly ileocolonic (57.89%) in patients with CD and pancolitis for patients with UC (56.25%). Most of the elderly IBD subjects were characterized by significant comorbidities, with Charlson Comorbidity Index (CCI) ≥ 1 in 66.67% patients. The probability of stopping biological therapy due to adverse events had the tendency to be higher in the CCI ≥ 1 group (20.58% vs. 5.88% in CCI = 0; *p* = 0.087). The main reasons for the therapy discontinuation included hypersensitivity reactions and liver enzyme abnormalities. **Conclusions**: In conclusion, our results underline the importance of assessing the comorbidity status instead of the age prior to initiating biological therapy, analyzing additional safety risks, and close monitoring in IBD patients with multiple comorbidities.

## 1. Introduction

Inflammatory bowel diseases (IBDs), such as ulcerative colitis (UC) and Crohn’s disease (CD), are chronic, progressive disorders, characterized by episodes of relapse and remission. Although IBD generally affects the young adult population, 10–15% of patients develop the disease after turning 60, constituting an extending group owing to the aging of the general population [1,2,3]. The elderly IBD patients pose challenges, especially due to the presence of age-related comorbidities and a possible greater diagnostic delay. It is known that physicians caring for this population must face several age-specific problems, including the safety of IBD therapy in the elderly population [4,5]. Nowadays, blocking the pathological pathways of the release of pro-inflammatory cytokines has become the focus of the current treatment of IBD. The introduction of biological therapies has influenced the management of IBD with a possible impact on their natural history, especially regarding the development of complications [6].

Previous studies focusing on elderly IBD patients suggested that the clinical features in these patients are different from those in the younger population [7,8,9]. However, there are scarce data concerning the most appropriate therapeutic approaches in this group of IBD patients. It is known that young patients are more biologically experienced than the elderly subjects, which may indicate the therapy-prescribing physician’s inclination to be more conservative in treating IBD in older patients [10,11]. Concerns over the safety of biological therapy in this population have been raised because of the patient’s advanced age and the significant comorbidity burden [12]. Moreover, data regarding biological therapy come mainly from clinical trials from which patients older than 65 are usually excluded [13]. Some studies reported that elderly patients treated with biological therapy were more likely to develop infections or malignancies and to be withdrawn from treatment more frequently as compared with a younger group [14,15,16]. However, the recent data also confirm the safety of biological therapy in an older group of IBD patients [17,18,19]. Furthermore, in real-world settings, biologic users with IBD are usually older in comparison to those treated in controlled settings, indicating the necessity of conducting detailed studies in the population of elderly patients with IBD [20,21].

The aim of our study was to assess the clinical characteristics of IBD patients over 60 years of age and to analyze the safety of biological therapy in this group of patients treated in two referral centers within the National Drug Program in Poland.

## 2. Methods

The data of all the patients treated with the biological therapy at some point between 2015 and 2022 were retrieved from the available local medical records of two centers in Poland (Department of Digestive Tract Diseases, Medical University of Lodz and Department of Gastroenterology, Konskie). Inclusion criteria were the receipt of biological treatment, age 60 years or over, and documentation of IBD diagnosis. The diagnoses of CD and UC were determined according to the endoscopic, radiological, and histological criteria [22,23]. The clinical history of IBD patients over 60 years of age was thoroughly analyzed. The retrospectively collected data included the type of IBD, gender, age at diagnosis, and presence of extraintestinal manifestations (EIMs). Moreover, the location and course of CD and UC were reported. EIM included anemia as well as joint, skin, ocular, and hepatobiliary manifestations. Comorbidity was assessed according to the Charlson Comorbidity Index (CCI), comprising 19 conditions listed in Table 1. Each reported disease is given a different weight based on the strength of its association with 1-year mortality where a CCI of 0 represents the absence of comorbidity [24]. In our analysis, as in other comparable studies, patients were divided into 2 groups: CCI = 0 and CCI ≥ 1, and we reported the safety of the biological therapy in IBD patients with the comparison of outcomes depending on CCI score.

All the previously used medications and surgeries conducted due to IBD were recorded. The patients were included in our study on the condition that they had received the biological therapy at some point between the years 2015 and 2022 according to the rules of the National Drug Program in Poland. In 2015–2017, only anti-tumor necrosis factor (anti-TNF-α) drugs were available: infliximab (IFX) for UC, as well as IFX and adalimumab (ADA) for CD. Since 2018, vedolizumab (VDZ), an α4β7 integrin antagonist, and since 2019, ustekinumab (UST), a monoclonal antibody to the p40 subunit of IL-12 and IL-23, have been reimbursed in Poland. Currently, in our National Drug Program IFX, VDZ and UST are available for UC therapy and ADA, IFX, VDZ (each as a first-line drug), as well as UST (in the second line of treatment after the failure of anti-TNF-α antibodies) for CD. The diagnosis of microscopic colitis, small molecules, and biologics for other reasons than IBD were exclusion criteria. The subjects with indeterminate IBD were not included in this analysis.

The adverse events collected for the safety analysis included infections, hypersensitivity reactions, liver test abnormalities (3× above the upper limit of the normal range), headache, myalgia, or cardiovascular events. Moreover, the need for surgery related to IBD, malignancies, major adverse cardiovascular events, or any other medical event that resulted in hospitalization were reported. The reasons for the discontinuation of the biological agent administration were also evaluated.

The study was performed as a clinical, retrospective study with anonymized data, and as such, it is exempt from the need for written informed consent. The study protocol was approved by the local ethics committee (nr RNN/293/23/KE).

## 3. Statistical Analysis

For the statistical analysis, the arithmetic averages were calculated. Qualitative data were expressed as numbers and percentages. For measurable features, the frequency of occurrence and the percentage share of individual categories in respective groups were evaluated. The significance of the differences between the studied groups was calculated using the Mann–Whitney U test and χ^2^ test. *p*-values < 0.05 were considered statistically significant. All the statistical calculations were performed using the Statistica 13.1 program by StatSoft, Inc. (Kraków, Poland).

## 4. Results

### 4.1. Demographic Characteristics of the Study Populations

The clinical data of 366 patients with CD or UC treated with biological agents were analyzed. Out of the entire group of the studied patients, 51 (13.9%)—24 men and 27 women—were aged over 60 years. The stratification of the elderly population according to age and gender or the type of the diagnosed IBD is shown in Table 2 and Table 3. In the group of the elderly patients, thirty-two (62.7%)—fifteen men and seventeen women—had been diagnosed with UC, and nineteen patients (37.3%)—nine men and ten women—with CD. The average age of elderly patients was 68.2 ± 4.3 years, with IBD diagnosed at an average age of 51.2 ± 6.3 years. The disease location was predominantly ileocolonic (57.89%) in patients with CD and pancolitis in patients with UC (56.25%). Proctitis was observed only in three (9.37%) patients with UC. Interestingly, we observed statistical significance in the context of proctitis; the frequency of proctitis in the UC-patients was higher in the subjects with CCI = 0 compared to those with CCI ≥ 1 (*p* = 0.039) (Table 4). Regarding the disease course, we observed the predominance of inflammatory pattern of CD—11 (57.89%) patients. A stricturing disease was present in five (26.32%) CD patients, and the remaining three (15.79%) patients had a penetrating type (Table 4). Among patients with CD, 26.32% suffered from perianal disease. Extraintestinal manifestations were diagnosed in almost half of the analyzed elderly IBD patients; anemia and articular manifestations were the most frequently observed (Table 2). Overall, 19 (37.2%) of the analyzed elderly patients had a history of at least one surgical resection related to IBD with no major differences between CD and UC patients, respectively: eight (42.13%) versus eleven (32.35%); *p* > 0.05.

The elderly patients in both the CD and UC groups had frequent cardiovascular (CD 31.58%; UC 34.38%; *p* = 0.418), metabolic (CD 15.79%; UC 18.74%; *p* = 0.392), and respiratory (CD 15.79%; UC 9.37%; *p* = 0.743) comorbidities. CCI of at least 1 was observed in 66.67% of elderly IBD patients, CCI of at least 2 in 27.45%, and of at least 3 in 5.88% (Table 2). A total of thirty-one (60.79%) patients received anti-TNF-α antibody therapy (IFX or ADA), while fourteen (27.45%) patients received VDZ, and six (11.66%) were treated with UST. In our study, there was a tendency to give VDZ more frequently to the patients with CCI ≥ 1 compared to those with CCI = 0 (*p* = 0.074), with no correlation regarding CCI status and other biologics, such as adalimumab (*p* = 0.387), infliximab (*p* = 0.117), and ustekinumab (*p* = 0.449). Most patients with a CCI ≥ 1 (61.76%) had no history of previous biological treatment. On the other hand, four patients (11.77%) with CCI ≥ 1 were treated with three biological drugs. The characteristics of the analyzed groups according to CCI score are summarized in Table 4.

### 4.2. Adverse Events and Safety

In 51 subjects, the frequencies of AE, including infections, hypersensitivity reactions, liver test abnormalities, headache, myalgia, and cardiovascular events were different, reaching 27.45%, 9.8%, 7.84%, 5.88%, 3.92%, and 3.92%, respectively. The most frequently observed adverse events in the elderly IBD patients were infections, including mild infections of the nasopharynx or urinary tract, which did not require hospitalization. There was also pneumonia, *Clostridioides difficile* infection, and interintestinal abscesses, all observed in the CCI ≥ 1 patients. Six (11.76%) patients required hospitalization for infectious complications, slightly more often in those with comorbidities (five patients (14.7%) CCI ≥ 1 vs. one patient (5.88%) with CCI = 0; *p* = 0.128). In the patients hospitalized for infections, *Clostridioides difficile* (two patients) and pneumonia (two patients) were the most common diagnoses, followed by urosepsis (one patient) and abscess (one patient). Other observed adverse events included an acute or delayed hypersensitivity reaction, liver enzymes abnormalities, cardiovascular events, and non-specific symptoms, such as a headache or myalgia. It is worth emphasizing that the percentage of adverse events, such as infections (32.35% vs. 17.64%; *p* = 0.134) and liver test abnormalities (8.83% vs. 5.88%; *p* = 0.356), was higher in the group with CCI ≥1 compared to the CCI = 0 group. In contrast, the percentage frequency of hypersensitivity reactions (11.76% vs. 8.83%; *p* = 0.37), myalgia (5.88% vs. 2.94%; *p* = 0.305), and cardiovascular events (5.88% vs. 2.94%; *p* = 0.305) was higher in the CCI = 0 group. Nevertheless, the number of patients in the group with CCI ≥1 was twice as big. Comparing this result to the most recent data, the risk of these AEs seems to be comparable to the elderly patients with IBD treated without using biological treatment and the elderly patients without IBD [25,26,27]. There were no differences in the rates of adverse events when the elderly population was stratified according to age. However, there was a slightly higher need for surgery related to IBD, excluding elective surgical treatment of perianal lesions, in the patients with comorbidities (five patients (14.7%) CCI ≥ 1 vs. one patient (5.88%) with CCC = 0; *p* = 0.128).

Moreover, the probability of terminating the biological therapy due to adverse events had the tendency to be higher in the CCI ≥1 group (20.58% vs. 5.88% in CCI = 0; *p* = 0.087). The main reasons for the therapy discontinuation were the hypersensitivity reactions and liver enzyme abnormalities (Table 5). The hypersensitivity reactions were observed in four patients during IFX therapy, in one patient treated with VDZ, and in none with ADA or UST. The frequency of the therapy’s discontinuation did not differ statistically depending on the drug’s type. In our study, the overall incidence of the majority of adverse events, including infections, was similar among elderly IBD patients regardless of the biological drug they were treated with (*p* > 0.05). In the presented study, there was no death and no new malignant neoplasms diagnosed during the treatment period apart from the diagnosis of basal cell skin cancer in one patient. There were no other significant differences in terms of adverse events between the analyzed group of elderly IBD patients.

## 5. Discussion

It was proven that the clinical manifestation of IBD in the elderly patients varies in comparison to the adult and young subjects, including non-specific presentation of the disease, smaller extent of lesions, or less risk of disease progression [8,9,28]. On the other hand, these patients are more likely to be malnourished, anemic and hypovolemic, with more frequent requirements of transfusion, and may need longer hospital stay [29]. Moreover, despite a suggestion of milder phenotype in IBD patients over 60 years of age, the rates of surgery and hospitalization are similar to those of the younger population. Furthermore, patients with elderly-onset IBD do not satisfactorily react to mesalamine or immunomodulators and increasingly often require biological treatment [2,15,16]. According to recent research, including that conducted among the Polish population, UC is more often diagnosed compared to CD. Also, in our study, the majority of the IBD elderly patients requiring biological therapy were diagnosed with UC [1,3]. From the endoscopic point of view, pancolitis and left-side colitis were dominant among our UC patients and CD course at diagnosis was inflammatory in approximately 58% of the elderly patients. Among the extraintestinal manifestations, anemia was observed most often both in the CD and UC patients, similar to our previous analysis [30].

In the presented study, most patients had cardiovascular, metabolic, or respiratory comorbidities with CCI ≥ 1. Comorbidity is one of the essential factors to be considered in any therapeutic decisions, not only in elderly patients. However, it has not been included systematically in any previous studies. In the research of Asscher et al., all the IBD patients aged 16 or older treated with VDZ or UST were enrolled prospectively with comorbidity assessment using CCI score. In patients who administered VDZ, there was a significant correlation between the CCI and the occurrence of infection during treatment, independent of age, gender, IBD type, concurrent medication, or disease duration. Moreover, the CCI, not age, was independently associated with the number of hospitalizations during the treatment in both the VDZ- and UST-treated patients. This study underlines the importance of comorbidity assessment in younger populations [31]. Similarly, it was found that the presence of comorbidity, especially of cardiovascular disease, was a better indicator of serious infections during anti-TNF-α antibody therapy compared to patients’ age [32]. In our research, some kinds of infections, including *Clostridioides difficille*, were also observed only in patients with CCI ≥ 1.

Of all the biologics, most data are available for anti-TNF-α antibody treatment and suggest the increased infectious complications in the IBD elderly patients [15,16,33]. In an Italian multicenter cohort of IBD patients aged above 65 years, 11% developed severe infections; moreover, 10% of those died because of them [16]. In a recently published study, Cheng et al. reported that among elderly IBD patients with CCI > 1 initiating biological therapy, anti-TNF-α treatment was associated with higher rates of infection-related hospitalizations than VDZ or UST therapy [33]. In addition, it was proven that the elderly patients treated with the anti-TNF-α antibodies have a higher rate of severe adverse events compared to the younger patients undergoing the same therapy [15]. The higher infection risk observed in some studies in the elderly population treated with the anti-TNF-α antibodies should probably be of special concern and the awareness of its presence in this group of patients should be heightened.

In the presented study, we did not observe any significant association between noted infections and the selected biological treatment. Likewise, in a recently published systematic review and meta-analysis of 15 studies with 1978 elderly IBD patients, the rates of infections did not differ no matter what biological drug was used. This meta-analysis included 841 patients treated with anti-TNF-α antibodies, 816 with VDZ and 321 with UST. As expected, it confirmed that the infusion or injection reactions were more common in the patients receiving the anti-TNF-α antibody treatment without any observed differences in the occurrence of other adverse events [12]. Conterminously, it was reported that the use of various biologics was burdened with a comparable risk regarding drug safety in the elderly IBD population and there was no possibility to create a preferred sequencing order among them [34].

However, VDZ theoretically has an advantage over other biological drugs due to the localized gut action mechanism and low impact on systemic immunosuppression [19,35]. Recent studies confirm the low risk of complications of VDZ therapy in elderly patients [19,35,36,37]. Other studies, including a retrospective analysis published by Sands et al., showed similar results concerning safety in the elderly CD patients treated with UST [35,38,39]. In the study of Holvoet et al., when comparing the safety profile of VDZ and UST treatment in the IBD elderly patients, no significant increase in the number of adverse events was observed [40]. On the other hand, Cohen et al. noted that the elderly IBD patients receiving VDZ have remarkably increased rates of upper respiratory infections compared to young IBD patients treated with the same drug. However, it is worth emphasizing that, in most cases, the infections did not result in therapy discontinuation; only 3.5% of the patients required discontinuation of the therapy due to adverse events [17].

Regarding the necessity of discontinuation of biological therapy, Desai et al. showed that the elderly patients treated with anti-TNF-α antibodies were withdrawn from the therapy more frequently compared to the younger population [15]. In our study, hypersensitivity reactions among the elderly IBD patients, especially during the IFX treatment, were the most frequent cause of the discontinuation of the therapy due to adverse events. Moreover, a greater necessity to discontinue the therapy was observed in the patients with CCI ≥ 1. Liver test abnormalities were especially observed in this group of patients.

It is also known that comorbidities are the leading causes of hospitalization in elderly IBD patients and older age is an independent risk factor for increased hospital fatality [15,16]. Apart from the development of basal cell skin cancer in one patient, no malignant neoplasms or death was observed during the analyzed treatment period. However, it is true that the worse outcomes of treatment in hospitalized elderly IBD patients and high mortality underpin the need for well-designed clinical trials for this population. In a real-life, multicenter study, the risk of developing malignancy was elevated in the patients older than 60 years of age previously treated with anti-TNF-α antibodies [40]. On the other hand, in a recently published meta-analysis, a higher rate of malignancies was observed in the elderly patients using VDZ or UST (mean rate: 2.14/100 pts years) [12]. However, the authors concluded that this result may represent a selection bias phenomenon; physicians are probably more likely to start UST or VDZ therapy in patients with a higher risk for malignancy based on the beneficial safety profile of the new biological drugs reported in clinical trials [12]. Lobaton et al. observed that age above 65 and CCI > 0 were independent risk factors of malignancy and mortality of IBD patients regardless of the medication [14]. Additionally, Asscher et al. did not show an association between the anti-TNF-α antibody treatment and the development of malignancies. Moreover, patient age did not affect the occurrence of malignancies; however, this risk was increased when multiple comorbidities were present [32].

The major limitation of our study is its retrospective nature, with potential biases including reporting bias of adverse events, especially of mild infections, which patients may not have reported. However, as the data in all the patients were obtained from medical reports, reporting bias would have affected all the IBD patients equally. Further limitations include the relatively small number of patients and incomplete clinical data, with the possibility that certain information was unavailable for comparison. Too few patients were included in this study to significantly conclude on the safety of biologics in the elderly subjects with IBD. In addition, reporting the adverse events could be unreasonable, because some of the included patients could have previous use of biological drugs, and thus possibly more exposure to the treatment. On the other hand, the strength of this study is primarily the collection of data from two centers in Poland, with the harmonized IBD biological treatment according to the National Drug Program.

## 6. Conclusions

Our results demonstrate that elderly subjects with IBD are a specific patient group that requires a unique approach to management of the therapy. Biological therapy seems to be safe. The choice of therapeutic option has a key significance for the clinical course of disease and depends on a lot of factors, mainly comorbidity. Further studies are necessary to determine detailed safety of biologics in elderly patients with IBD, as well as develop adequate schemes of therapy using biological drugs in this group of the patients.

## Figures and Tables

**Table 1:** Charlson Comorbidity Index (CCI).

Comorbidities	Score
Metastatic solid tumor AIDS	6
Moderate or severe liver disease	3
Hemiplegia Moderate or severe renal disease Diabetes with chronic complications Tumor without metastases Leukemia Lymphoma	2
Prior myocardial infarction Congestive heart failure Peripheral vascular disease Cerebrovascular disease Dementia Chronic pulmonary disease Connective tissue disease Peptic ulcer disease Mild liver disease Diabetes	1

**Table 2:** Baseline characteristics of 51 patients.

Variables	CD (n = 19)	UC (n = 32)	Total (n = 51)
Age			
60–65	7 (36.84%)	9 (28.13%)	16 (31.37%)
65–70	7 (36.84%)	10 (31.25%)	17 (33.33%)
70–75	4 (21.05%)	8 (25%)	12 (23.53%)
75–80	1 (5.27%)	5 (15.62%)	6 (11.77%)
>80	0 (0%)	0 (0%)	0 (0%)
EIM			
Anemia	8 (42.11%)	13 (40.63%)	21 (41.18%)
Articular	8 (42.11%)	5 (15.63%)	13 (25.49%)
Cutaneous	1 (5.26%)	3 (9.38%)	4 (7.84%)
Ocular	1 (5.26%)	1 (3.13%)	2 (3.92%)
PSC	0 (0%)	1 (3.13%)	1 (1.96%)
CCI			
0	7 (36.84%)	10 (31.25%)	17 (33.33%)
1	8 (42.11%)	12 (37.5%)	20 (39.22%)
2	4 (21.05%)	7 (21.88%)	11 (21.57%)
≥3	0 (0%)	3 (9.38%)	3 (5.88%)
Previous biologics			
0	11 (57.89%)	18 (56.25%)	29 (56.86%)
1	5 (26.32%)	9 (28.13%)	14 (27.45%)
2	3 (15.79%)	4 (12.5%)	7 (13.73%)
3	0 (0%)	1 (3.12%)	1 (1.96%)

**Table 3:** Characteristics of elderly patients with IBD depending on age and sex.

Age	Women (n = 27)	Men (n = 24)	Total (n = 51)
60–65	7 (25.93%)	9 (37.5%)	16 (31.37%)
65–70	8 (29.63%)	9 (37.5%)	17 (33.33%)
70–75	7 (25.93%)	5 (20.83%)	12 (23.53%)
75–80	5 (18.52%)	1 (4.17%)	6 (11.77%)
>80	0 (0%)	0 (0%)	0 (0%)

**Table 4:** Demographic characteristics of patients > 60 years old depending on CCI.

	Total n = 51	CCI = 0 n = 17 (33.33%)	CCI ≥ 1 n = 34 (66.67%)	*p*
Sex, female n (%)	27 (52.94%)	9 (52.94%)	18 (52.94%)	0.50
Age in years ± SD	68.2 ± 4.1	65.1 ± 3.7	69.7 ± 4.3	0.351
IBD type				
UC	32 (62.74%)	10 (58.82%)	22 (64.71%)	0.341
CD	19 (37.26%)	7 (41.18%)	12 (35.29%)	0.341
CD behavior				
Inflammatory	11 (21.57%)	5 (29.41%)	6 (17.65%)	0.072
Stricturing	5 (9.8%)	1 (5.88%)	4 (11.76%)	0.074
Penetrating	3 (5.88%)	1 (5.88%)	2 (5.88%)	0.413
CD perianal disease	5 (9.8%)	2 (11.76%)	3 (8.82%)	0.392
UC location				
Pancolitis	18 (35.29%)	6 (35.29%)	12 (35.29%)	0.355
Left-sided	11 (21.57%)	2 (11.76%)	9 (26.47%)	0.069
Proctitis	3 (5.88%)	2 (11.76%)	1 (2.94%)	0.039
Number of Biological use				
1	29 (56.86%)	8 (47.06%)	21 (61.76%)	0.159
2	14 (27.45%)	5 (29.41%)	9 (26.47%)	0.412
3	7 (13.73%)	3 (17.65%)	4 (11.77%)	0.283
4	1 (1.96%)	1 (5.88%)	0 (0%)	
Biological (first line)				
Adalimumab	7 (13.73%)	2 (11.76%)	5 (14.70%)	0.387
Infliximab	24 (47.06%)	10 (58.83%)	14 (41.18%)	0.117
Ustekinumab	6 (11.76%)	2 (11.76%)	4 (11.77%)	0.499
Vedolizumab	14 (27.45%)	3 (17.65%)	11 (32.35%)	0.074

**Table 5:** Adverse events (AEs) and discontinuation of the therapy due to AE in the analyzed group of patients > 60 years old depending on CCI score.

	Total n = 51	CCI = 0 n = 17 (33.33%)	CCI ≥ 1 n = 34 (66.67%)	*p*
Infections	14 (27.45%)	3 (17.64%)	11 (32.35%)	0.134
nasopharyngeal	4 (7.84%)	2 (11.76%)	2 (5.88%)	0.231
urinary tract	4 (7.84%)	1 (5.88%)	3 (8.83%)	0.356
Clostridioides difficile	2 (7.84%)	0	2 (5.88%)	
pneumonia	2 (3.92%)	0	2 (5.88%)	
other	2 (3.92%)	0	2 (5.88%)	
Hypersensitivity reactions	5 (9.8%)	2 (11.76%)	3 (8.83%)	0.370
Liver test abnormalities	4 (7.84%)	1 (5.88%)	3 (8.83%)	0.356
Headache	3 (5.88%)	1 (5.88%)	2 (5.88%)	0.500
Myalgia	2 (3.92%)	1 (5.88%)	1 (2.94%)	0.305
Cardiovascular events	2 (3.92%)	1 (5.88%)	1 (2.94%)	0.305
Therapy discontinuation	8 (15.68%)	1 (5.88%)	7 (20.58%)	0.087
hypersensitivity reactions	4 (7.84%)	1 (5.88%)	3 (8.83%)	0.356
liver test abnormalities	2 (3.29%)	0	2 (5.88%)	
urine sepsis	1 (1.96%)	0	1 (1.96%)	
other	1 (1.96%)	0	1 (1.96%)	

## Data Availability

The data presented in this study are available on request from the corresponding author.

## Abbreviations

ADA, adalimumab; CCI, Charlson Comorbidity Index; CD, Crohn’s disease; IBD, inflammatory bowel disease; IFX, infliximab; UC, ulcerative colitis; UST, ustekinumab; VDZ, vedolizumab.
